# Substrate Marking by an Invasive Ladybeetle: Seasonal Changes in Hydrocarbon Composition and Behavioral Responses

**DOI:** 10.1371/journal.pone.0061124

**Published:** 2013-04-04

**Authors:** Delphine Durieux, Bérénice Fassotte, Maryse Vanderplanck, Jean-Louis Deneubourg, Christophe Fischer, Georges Lognay, Eric Haubruge, François J. Verheggen

**Affiliations:** 1 Unit of Functional and Evolutionary Entomology, Gembloux Agro-Bio Tech, University of Liege, Gembloux, Belgium; 2 Laboratory of Zoology, University of Mons, Mons, Belgium; 3 Unit of Social Ecology, Université libre de Bruxelles, Brussels, Belgium; 4 Unit of Analysis Quality and Risk, Laboratory of Analytical Chemistry, Gembloux Agro-Bio Tech, University of Liege, Gembloux, Belgium; INRA-UPMC, France

## Abstract

The multicolored Asian ladybeetle, *Harmonia axyridis* (Pallas), aggregates inside dwellings during the winter to survive the cold. Recent published reports have highlighted that overwintering individuals use hydrocarbon markings deposited on surfaces by conspecifics to orient toward aggregation sites. In the current study, monthly GC-MS analyses revealed seasonal modifications in the chemical profile of substrate markings deposited by moving individuals. The markings of overwintering ladybeetles contained larger proportions of heptacosadiene, nonacosadiene, hentriacontadienes, and methyl-nonacosanes, along with a lower proportion of heptacosene and nonacosene. This finding suggests the importance of the unsaturated and/or branched hydrocarbons in the *H. axyridis* aggregation process. Subsequently, we conducted behavioral assays to test whether (1) there is seasonal variation in the behavioral response of *H. axyridis* individuals toward substrate markings deposited by conspecifics in the same physiological state and (2) the observed behavioral modification is due to a change in ladybeetle sensitivity and/or a change in the chemical composition of the substrate marking. The results indicate that overwintering individuals exhibit a stronger “following” response toward conspecific substrate markings. This behavior is linked to both the physiological state of ladybeetles and the specific chemical profile of the marking biomolecules deposited under overwintering conditions.

## Introduction

The multicolored Asian ladybeetle, *Harmonia axyridis* (Pallas), has become an interesting model to study insect aggregation behavior, with individuals of this species only forming visible aggregations during the cold season. This ladybeetle species aggregates inside dwellings and buildings to survive the cold, resulting in occupants becoming disturbed by stains, unpleasant odors, and allergic reactions [1]. Recent investigations have shown that non-volatile compounds play a key role as chemical signals that induce the aggregation of this household invader [2]. Substrate markings, composed of saturated and unsaturated hydrocarbons, have been observed both inside and outside aggregation sites. These markings have two different roles, namely to ensure the cohesion of aggregations and to lead arriving conspecifics toward aggregation, respectively. We focused our work on the second marking, which probably originates from the footprints of individuals walking on household surfaces [3]. Because *H. axyridis* only presents obvious aggregation behavior during overwintering to avoid unfavorable conditions, we hypothesized that ladybeetle behavior changes towards these conspecific chemicals according to the period of the year. Indeed, previous research has reported that the production of chemical substances, as well as the behavioral response toward these signals, varies according to the physiological state of the signaler or receiver, respectively [4], [5].

In this work, we evaluated the modifications of chemical profile in the substrate marking deposited by moving *H. axyridis* over a one-year period. In addition, we observed the behavioral impact of these deposited chemical cues on both male and female individuals. Our data provide evidence for an adaptive response of *H. axyridis* to substrate markings deposited by moving conspecifics, which is associated with both individual physiological state and the chemical composition of the signal in question.

## Materials and Methods

### Biological material

#### Collection of wild ladybeetles

From February 2011 to January 2012, about 200 ladybeetles were collected each month in the vicinity of Gembloux (Belgium). No specific permits were required for the described field studies, as H. axyridis is not an endangered or protected species. During the winter period (from October to March), specimens were collected from infested dwellings, including inside window and door frames, or on walls and windows, where individuals had aggregated to overwinter. However, in March, ladybeetles were no longer aggregated and were found walking around the vicinity of the overwintering site. During the other months (i.e., the summer months from April to September), specimens were collected from the outside environment, inhabiting a diverse range of plant species, including lime trees, chestnut trees, maple trees, and blackberry bushes. Both outdoor and indoor collections were performed by brushing ladybeetles delicately into small containers with a pencil. All individuals were adults, which were subsequently placed in 36×15×8 cm aerated plastic boxes(±40 individuals per container) throughout the tests, which lasted a maximum of one week. To preserve the physiological state of individuals collected outside (from April to September), the boxes were placed on a window sill in a non-heated room to replicate outdoor conditions. During the summer months (i.e., April to September), specimens in each box were provided with sugar, a water-impregnated sponge and bee-collected multiflower pollen. In contrast, individuals collected during winter (i.e., October to March) were placed in the dark at 15±1°C without any food or water, to replicate aggregation conditions. This temperature was in accordance with the mean temperature recorded at the aggregation sites investigated during this study (14.37±3.66°C, n = 10).

About 100 aggregated ladybeetles were collected from a window frame in October 2012, with the aim of using them in a negative control. These insects were maintained under the same conditions as individuals collected during the previous winter until and throughout the tests which lasted no longer than one week.

#### Ladybeetle rearing

Rearing was set up using H. axyridis individuals collected in the vicinity of Gembloux during the winter of 2008. These adults were placed in 36×15×8 cm aerated plastic boxes(±20 individuals per container), and provided with sugar, a water-impregnated sponge and bee-collected multiflower pollen. The boxes were placed in a controlled environment chamber with a 16 h-light photoperiod, 24±1°C temperature, and 45±15% relative humidity (RH). Oviposition was induced by the introduction of aphids Acyrthosiphon pisum Harris and the resultant larvae were provided with a daily supply of A. pisum. These aphids were mass-produced on Vicia faba plants, which were grown in a 30×20×6 cm plastic tub containing a mixture of vermiculite and perlite (1/1). Both A. pisum and V. faba were kept under the following conditions: 16-h light photoperiod, 20±2°C temperature, and 60±5% RH.

The rearing H. axyridis used in the tests belonged to the eighth generation. Each previous generation had a minimum population of 50 individuals to avoid degeneration due to consanguinity. All generations were maintained under the previously stated conditions for photoperiod, temperature, and RH. The eighth generation was obtained after 2 years.

### Collection of substrate marking

We collected the markings deposited by the specimens that were collected each month, while they were in motion (in comparison, during the overwintering period, to individuals having formed an aggregation and do not move anymore). To do so, 40 ladybeetles were placed in a glass Petri dish (diameter: 18 cm, height: 4 cm) that had been previously cleaned with the liquid detergent RBS T 105 (Chemical Products R. Borghgraef, Brussels, Belgium) and norvanol (ether-denatured ethanol; VWR International, Haasrode, Belgium). The beetles were then allowed to mark the surface by moving about for a 24-h period. We did not differentiate between males and females when obtaining the markings, as the cuticular profile of *H. axyridis* is the same for both sexes (personal observation). Four replicates were conducted for each monthly collection. These Petri dishes were kept at 24±1°C, 45±15% RH and at a luminosity of 1600±200 lux, under a photoperiod replicating that observed during each monthly collection. After 24 h in the Petri dish, the ladybeetles were removed and the markings deposited, either on the base or on the cover of each Petri dish, were collected with approximately 0.15 g of quartz wool (fiber size of 2–12 µm; FilterService S.A., Eupen, Belgium), which had been impregnated with 150 µl of n-hexane (HPLC grade, 95% of purity; Fisher Scientific, Loughborough, Leicestershire UK). The chemicals collected from each Petri dish (both base and cover) were dissolved in 950 µl of n-hexane. To avoid feces being deposited onto the Petri dishes, non-wintering individuals were starved for 24 h before being placed in the Petri dishes. All extracts were kept at −18°C until chemical analyses and then until their use in a bioassay.

### Chemical analyses

#### Identification of compounds

The chemical profile of the four replicates was compared for each monthly collection by injecting 1 µl of every replicate onto a Thermo Trace GC Ultra (Thermo Electron Corporation) (cf section “Quantitative variation in chemical profile” below). If the chemical profile was similar for all four injections, the samples were pooled and the identification of the collected compounds was carried out on the resultant extract using gas chromatography-mass spectrometry (GC-MS). The analyses were carried out by injecting 1 µl of each pooled n-hexane extract onto a Thermo Trace GC Ultra equipped with an Optima 5-MS Accent column (30 m x 0.25 mm I.D.; film thickness 0.25 µm) coupled to a Thermo Trace MS Plus (Thermo Electron Corporation). The operating conditions were: splitless, injector at 280°C; carrier gas: helium at 1.0 ml/min; temperature program: from 40°C (held during 2 min) to 320°C at 10°C/min, with a final hold of 3 min at 320°C. The mass spectra were recorded in the electron impact mode at 70 eV (source temperature at 230°C, transfer line at 250°C, scanned mass range: 40 to 450 m/z). The detected peaks were identified from their retention data and their characteristic fragmentation patterns. The identification of saturated compounds was then confirmed by the injection of standard n-alkanes (from C_9_ to C_40_). The identification of the methyl position in branched alkanes was determined from the occurrence of C_n_H_2n+1_ and C_n_H_2n_ fragments after α-cleavage at the branch positions [6].

To determine the double bond position of monounsaturated compounds, epoxidation using m-chloroperbenzoic acid was performed [7]. Each epoxide was then characterized by two specific fragments in the mass spectrometer [8]. This reaction was performed on approximately 500 µg of collected material recovered from the n-hexane solution. The n-hexane was evaporated at 50°C in a Büchi Rotavapor R-114, then 200 µl of chloroform (99% purity; Merck KGaA, Darmstadt, Germany) and 200 µl of a chloroformic solution of m-chloroperbenzoic acid (25 mg/ml) (*p.a.* Acrōs Organics, New Jersey, USA) were added to the residue. The resulting blend was continuously agitated for 2 h. Subsequently, 200 µl of an aqueous solution of sodium bisulfite (50 mg/ml) (*p.a.* Acrōs Organics) and sodium bicarbonate (50 mg/ml) (*p.a.* Carlo Erba, Milano, Italy) were added, followed by 1 ml of chloroform. The resulting mixture was rinsed twice with 1 ml distilled water and dried using anhydrous sodium sulfate (Merck KGaA, Darmstadt, Germany). Finally, the sample was kept at −18°C until GC-MS analysis. Unfortunately, this reaction did not allow us to identify the position of the double bonds in alkadienes because of the recombination of the formed fragments [7].

To verify that the double bond position of monoenes is stable throughout the year, epoxidation was performed on half of the collected extracts, specifically the markings obtained during February, April, June, August, October and December.

#### Quantitative variation in chemical profile

To assess the quantitative seasonal modification in the chemical profile of markings, we performed the quantification of the collected amounts of each component. This step was carried out on each replicate using gas-liquid chromatography (GLC) on a Thermo Trace GC Ultrafast (Thermo Electron Corporation) equipped with a flame ionization detector (FID at 310°C) (300 Hz) and a Ph5 column (5 m×0.1 mm×0.1 µm). The injections were performed with a split ratio of 20:1. The injector temperature was 310°C, and the carrier gas was helium (0.5 ml/min). The temperature program was 40°C for 30 s, then an increase of 60°C/min to 310°C, which was held for 1 min. Compounds were quantified with n-nonadecane as the internal standard (IS) (22 µg/ml), because this compound was absent in the extracts. Given that all chemicals could not be fully identified, the differential FID response to the various compounds could not be taken into account. Four replicates were performed for each month.

#### Statistical analyses

To visually assess the differences in the chemical composition of substrate markings across months, we performed a non-metric multidimensional scaling (NMDS) ordination, which was based on the Bray-Curtis distance. Statistics were conducted in R using functions from ecodist, ellipse (based on standard deviation) and BiodiversityR. All NMDS plots were generated employing a Bray-Curtis similarity matrix, two dimensions (applying a conventional cutoff of <0.2 for the stress value) and 50 runs to more fully explore the ordination space at that dimensionality. The resulting minimum stress solution was used to produce the NMDS plots, in which each data point represents one sample. The spatial distance between points in the plot may be interpreted as the relative difference in the composition of substrate marking. Hence, points that are closer are more similar than points that are more distant.

To analyze the differences between the resulting groups, perMANOVA (“adonis” command, R-package vegan) and an indicator compound analysis (“indval” function, R-package labdsv) were performed. PerMANOVA is a permutation-based version of the multivariate analysis of variance (MANOVA). Like conventional analyses of variance, perMANOVA calculates an F statistic from the ratio of the between-group sum of squares and the within-group sum of squares. Since it is non-parametric, this analysis does not have to meet particular assumptions. The indval analysis allowed us to identify the indicator compounds of one group. In other words, this analysis allowed us to identify the compounds that were simultaneously present and abundant in one group compared to the other groups. This analysis produced an indicator value for each compound. In addition, a *P*-value was calculated for each compound-group combination, to assess whether compounds were significant indicators of certain months. All multivariate data visualization and analyses were conducted in R version 2.9.1.

### Bioassays

The behavior of *H. axyridis* individuals toward substrate markings deposited by conspecifics in the same physiological state (i.e., insects collected at the same time) was observed over a 1-year period in a Y-shaped glass tube (length: 10 cm, diameter: 1 cm) to highlight any potential seasonal change in ladybeetles response. Such a change might be due to (1) an increase in the sensitivity of overwintering ladybeetles toward the markings deposited by conspecifics and/or (2) a modification in the leading potential (i.e., the potential inducing a “following” response in ladybeetles) of the substrate marking. To assess the possible contribution of these factors separately, two additional experiments were performed using the same Y-shaped device. The first hypothesis was tested by evaluating the behavioral response of wild *H. axyridis* individuals collected each month toward a standard winter substrate marking (see Table 1 for the chemical profile of this marking). This reference extract had been obtained by using ladybeetles collected in dwellings situated in the vicinity of Gembloux during the winters of 2009 and 2010 (from October to February). The markings deposited by these overwintering *H. axyridis* had been collected and dissolved in 1 ml of n-hexane, by using the same protocol as that described in section “Collection of substrate marking”. During the 24-h period of marking, the Petri dishes, where ladybeetles had been previously introduced, had been placed at 22±1°C temperature. The extract used as reference resulted from the pooling of 5 replicates and was kept at −18°C. The leading potential of this reference extract had been demonstrated on overwintering individuals in a previous study [2].

**Table 1:** Chemical profile (mean % of each compound±CI, calculated by relating individual peak areas to the internal standard peak area, n = 4) of (1) substrate markings laid by moving *H. axyridis* adults in the month of the year that they were collected and (2) the reference winter extract involved in the bioassay testing the potential seasonal changes in ladybeetle sensitivity.

	Diagnostic ions (m/z)		Relative concentrations												
Identified compounds	molecule	epoxide	February^(a)^	March^(b)^	April^(c)^	May^(c)^	June^(c)^	July^(c)^	August^(c)^	September^(c)^	October^(a)^	November^(a)^	December^(a)^	January^(a)^	reference winter extract
**nC_23_**	324		13.15 ± 0.37	11.12 ± 0.03	10.71 ± 0.10	10.03 ± 0.48	10.72 ± 0.11	11.38 ± 0.27	9.75 ± 0.55	11.91 ± 0.40	13.36 ± 0.53	13.94 ± 0.35	11.84 ± 0.29	15.94 ± 0.19	14.91
**nC_24_**	338		0.39 ± 0.01	0.32 ± 0.01	0.34 ± 0.02	0.34 ± 0.02	0.41 ± 0.02	0.50 ± 0.04	0.57 ± 0.06	0.67 ± 0.02	0.47 ± 0.02	0.38 ± 0.01	0.33 ± 0.02	0.43 ± 0.02	0.41
**C_25_:1**	350	155-253	17.27 ± 0.30	23.04 ± 0.30	22.62 ± 0.73	20.39 ± 2.62	20.55 ± 0.83	16.65 ± 0.82	11.33 ± 1.70	20.37 ± 1.18	16.03 ± 0.84	15.18 ± 0.49	14.27 ± 0.54	15.67 ± 0.65	16.83
**nC_25_**	352		5.48 ± 0.06	6.33 ± 0.02	7.01 ± 0.16	6.62 ± 0.21	7.11 ± 0.19	6.73 ± 0.19	6.89 ± 0.22	7.67 ± 0.67	6.92 ± 0.19	4.71 ± 0.26	3.89 ± 0.22	5.27 ± 0.15	5.64
**C_27_:2**	376		2.72 ± 0.13	1.08 ± 0.03	< LOD	< LOD	< LOD	< LOD	< LOD	< LOD	1.51 ± 0.13	3.07 ± 0.21	3.51 ± 0.11	4.74 ± 0.27	2.72
**C_27_:1**	378	155-281	18.93 ± 0.76	26.54 ± 0.14	33.63 ± 0.36	31.38 ± 0.78	30.37 ± 0.15	27.05 ± 0.47	29.35 ± 0.60	22.45 ± 0.41	17.76 ± 1.11	14.08 ± 0.11	12.57 ± 0.34	11.83 ± 0.25	16.63
**nC_27_**	380		2.28 ± 0.13	3.54 ± 0.03	5.42± 0.31	4.19 ± 0.67	4.73 ± 0.21	1.69 ± 0.03	3.65 ± 0.25	2.21 ± 0.06	2.43 ± 0.13	1.45 ± 0.06	1.35 ± 0.10	1.64 ± 0.05	1.9
**C_29_:2**	404		16.15 ± 0.54	6.60 ± 0.22	0.24 ± 0.02	1.17 ± 0.62	0.74 ± 0.15	2.88 ± 0.16	2.15 ± 0.34	1.72 ± 0.30	13.26 ± 0.86	19.82 ± 0.31	21.83 ± 0.60	21.86 ± 0.33	16.94
**C_29_:1**	406	155-309	7.98 ± 0.18	8.69 ± 0.03	8.51 ± 0.14	14.28 ± 2.82	12.22 ± 0.88	18.96 ± 0.34	20.18 ± 1.52	15.17 ± 0.59	9.27 ± 0.28	7.67 ± 0.14	8.26 ± 0.25	5.56 ± 0.13	7.24
**nC_29_**	408		0.94 ± 0.02	0.90 ± 0.02	0.95 ± 0.10	0.87 ± 0.19	1.11 ± 0.08	0.49 ± 0.05	0.94 ± 0.11	0.60 ± 0.08	1.19 ± 0.05	1.00 ± 0.07	0.67 ± 0.04	0.99 ± 0.06	0.85
**13- &15-Me-C_29_**	196-224-252-407		1.32 ± 0.09	< LOD	< LOD	0.48 ± 0.29	< LOD	0.93 ± 0.10	1.22 ± 0.44	0.70 ± 0.23	0.97 ± 0.04	1.47 ± 0.09	1.58 ± 0.03	1.66 ± 0.04	1.31
**C_31_:2**	432		10.17 ± 0.44	4.88 ± 0.19	0.31 ± 0.05	2.85 ± 1.45	2.88 ± 0.60	9.39 ± 0.22	9.12 ± 1.14	10.23 ± 0.99	14.06 ± 1.00	15.05 ± 0.28	17.92 ± 0.33	12.78 ± 0.64	12.09
**C_31_:1**	434	155-337	3.23 ± 0.15	6.95 ± 0.08	10.27 ± 0.46	7.38 ± 1.91	9.16 ± 0.35	3.35 ± 0.34	4.86 ± 0.91	6.31 ± 0.55	2.77 ± 0.22	2.19 ± 0.12	2.00 ± 0.12	1.64 ± 0.03	2.54

Months during which ladybeetles were collected from aggregation sites.

a Month during which ladybeetles were collected when walking around in the vicinity of aggregation sites.

b Months during which ladybeetles were collected outside.

LOD, Limit of detection.

On the other hand, possible seasonal changes in the substrate marking potential were studied by testing 2–3 month old rearing ladybeetles (i.e., individuals in the same non-overwintering physiological state) toward the marking laid by *H. axyridis* individuals collected each month. These laboratory insects had never mated before the beginning of the experiment. It would be more appropriate to use ladybeetles in an overwintering state to highlight any modification in the leading potential of the substrate marking. We attempted to obtain in our rearing *H. axyridis* individuals in a state of reproductive diapause; however, like Berkvens et al. [9], we did not succeed. This is explained by the fact that the factors involved in the induction of this state are still misunderstood. It was therefore impossible for us to assess overwintering ladybeetles throughout the year.

Prior the beginning of the bioassays, the Y-shaped device was completely cleaned with RBS T 105 and norvanol. Thereafter, one of the two arms was coated with either 700 µl of the pooled extract of substrate marking deposited by ladybeetles collected each month, or 800 µl of the reference winter extract. The quantities used in the experiment replicated the amounts that were collected under natural conditions. These extracts were deposited using a 250 µl syringe (Hamilton, Reno, USA) in such a way that one arm and the base of the Y-shaped tube were uniformly marked. The selection of one of the two arms for individual male and female ladybeetles was then observed (n = 50 males; n = 50 females), the Y tube being placed horizontally. Each insect was introduced in the set-up by placing it gently at the base entrance with tweezers. The order in which males and females were tested was randomly defined for each bioassay. The experiment lasted three minutes, which was sufficient for the majority of individuals to explore the entire set-up. The response was considered positive when the ladybeetle passed the bend of the marked arm. The response was considered negative when the individual entered beyond bend of the unmarked arm. The response was considered as no-response when the ladybeetle remained in the base for the entire three minutes. The tube was cleaned and remarked once, after testing 50 individuals. Moreover, to verify that the chemicals laid by the tested ladybeetles did not interfere with the deposited marking, Chi-square Goodness of Fit Tests (Minitab® 15.1.1.0; α = 0.05, 1 df) were performed to compare the behavior of the first half of the tested individuals with that of the second half. To avoid any directional bias, the direction of the marked arm was switched after each replicate. Each time a ladybeetle chose the side free of chemical compounds, the arm was cleaned with norvanol in order to avoid the selection of subsequent individuals being influenced. Control assays, conducted in a Y-shaped glass tube, with one arm coated with norvanol, were first performed to verify that this solvent has no effect on the behavior of *H. axyridis*. All experiments were carried out from 9:00 to 17:00 in a growth chamber at 24±1°C temperature, 45±15% RH, and at a luminosity of 1000±200 lux.

Finally, a negative control was performed to study the behavior that a summer extract induced in overwintering ladybeetles. A reference summer extract, composed of 350 µl of the marking collected in July 2011 and 350 µl of the marking collected in August 2011, was placed in the Y-shaped glass tube. Once again, the behavior of 50 males and 50 females was observed. The tested ladybeetles were collected in October 2012. Furthermore, the response of these individuals to a winter marking had not been previously tested; hence, the same test was repeated on these ladybeetles (50 males and 60 females) toward 700 µl of the extract collected in January 2012. These two additional bioassays were performed with the same protocol described in this section.

For each assay, a Chi-square Goodness-of-fit Test (Minitab® 15.1.1.0; α = 0.05, 1 df) was used to compare the observed and expected frequencies (i.e., 50% of ladybeetles choosing each arm). Subsequently, a Chi-square Test of Independence (Minitab® 15.1.1.0; α = 0.05, 1 df) was performed to compare the behavior of males versus females. These tests were carried out on the observed absolute numbers. Ladybeetles that did not select one of the arms within three minutes were not included in the statistical tests.

## Results

### Seasonal changes in the chemical profile of substrate marking

**Figure 1:**
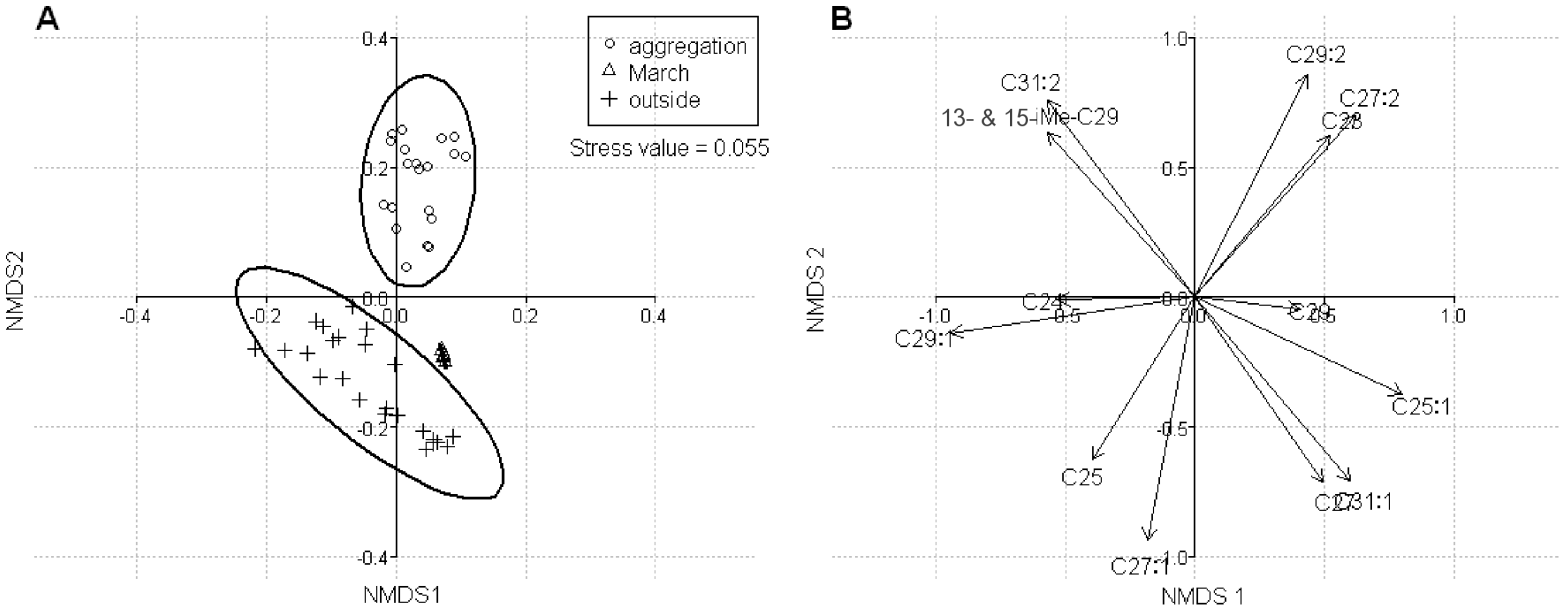
Non-metric multidimensional scaling (NMDS) plot. NMDS ordination of the chemical profile of substrate markings for axis 1 (NMDS 1) and axis 2 (NMDS 2) according to the physiological state of the individuals that deposited it (stress value = 0.055). The group “aggregation” brings together the substrate markings deposited by overwintering ladybeetles, the group “outside” gathers the substrate markings deposited by ladybeetles collected outside (i.e., from plants) and finally, the group “March” corresponds to the deposits from individuals caught in March (individuals were not aggregated, but were found walking around in the vicinity of the overwintering site).

The GC-MS analyses revealed that the extracts were composed of 13 compounds, which were identified as saturated and unsaturated hydrocarbons (Table 1). Two hentriacontadienes were present in the extracts; however, they could not be clearly distinguished in fast-GC chromatograms. They were then considered as only one compound. Table 1 shows the variation in chemical composition of substrate markings deposited by *H. axyridis* individuals for each month across a 1-year period. To reduce inter-replicate variation, only the chemical profile of the extracts was considered, with the deposited amounts being presented as percentages. NMDS ordination was performed on these percentages and arranged the chemical profile of the substrate markings along the second axis (NMDS 2) into two distinctive groups. The first group gathers the substrate markings deposited by individuals removed from aggregation sites (aggregation group) while the second group brings together the substrate markings deposited by *H. axyridis* collected in nature (outside group) (Fig. 1). The substrate markings from March did not fit into either group, instead being located midway between the two (March group). This result was further supported by perMANOVA, which detected a significant difference in the chemical profile between the three groups (*F* = 60.45, *P*<0.001 ***). Indicator Compound Analysis showed that heptacosadiene (indicator value = 74.22, *P* = 0.001), methyl-nonacosanes (indicator value = 71.62, *P* = 0.001), nonacosadiene (indicator value = 69.68, *P* = 0.001), hentriacontadienes (indicator value = 56.72, *P* = 0.001), and, to a lesser extent, tricosane (indicator value = 38.42, *P* = 0.004) were significantly associated with substrate markings deposited by aggregation individuals. Pentacosene was associated with the extracts collected in March (indicator value = 40.16, *P* = 0.003), while nonacosene (indicator value = 47.52, *P* = 0.002), heptacosene (indicator value = 41.13, *P* = 0.009), and, to a lesser extent, tetracosane (indicator value = 39.62, *P* = 0.017) and pentacosane (indicator value = 37.69, *P* = 0.021) were identified as indicator compounds for substrate markings deposited by *H. axyridis* collected outside. The statistical results of the Indicator Compound Analysis supported the ordination map (Fig. 1).

### Modification of H. axyridis behavior

The first Y-shaped tube bioassay offered single *H. axyridis* individuals the choice between an arm coated with substrate marking deposited by conspecifics in the same physiological state versus a clean arm. This assay demonstrated that all overwintering individuals (from October to February), except ladybeetles collected in November, preferentially chose the marked arm (Table 2). This preference was also observed for individuals in March and April, and for males collected in May and June (Table 2). The response of the ladybeetles collected each month toward the reference winter extract was exactly the same as toward the substrate marking deposited by the conspecifics with which they had been collected except in May, June and December, for which no preferential behavior was observed for males (Table 3).

**Table 2:** Results of the bioassay used to test the seasonal response of *H. axyridis* individuals toward substrate marking deposited by conspecifics in the same physiological state (n = 50 males; n = 50 females).

		Percentage of ladybeetles that selected the arm		Goodness-of-fit test statistical data		Test of independence statistical data	
Month	Sex	marked	clean	χ^2^	P	χ^2^	P
February	male	76	24	13.52	<0.001***	1.00	0.317
	female	84	16	23.12	0.001**		
March	male	78	22	15.68	<0.001***	0.22	0.640
	female	74	26	11.52	0.001**		
April	male	76	24	13.52	<0.001***	<0.001	>0.999
	female	76	24	13.52	<0.001***		
May	male	66	34	5.12	0.024*	2.03	0.155
	female	52	48	0.08	0.777		
June	male	66	34	5.12	0.024*	1.50	0.221
	female	54	46	0.32	0.572		
July	male	50	50	0.00	>0.999	0.36	0.548
	female	44	56	0.72	0.396		
August	male	42	58	1.28	0.258	0.16	0.687
	female	46	54	0.32	0.572		
September	male	54	46	0.32	0.572	0.04	0.841
	female	52	48	0.08	0.777		
October	male	64	36	3.92	0.048*	0.04	0.834
	female	66	34	5.12	0.024*		
November	male	42	58	1.28	0.258	<0.001	>0.999
	female	42	58	2.00	0.157		
December	male	70	30	8.00	0.005**	0.20	0.656
	female	74	26	11.52	0.001**		
January	male	72	28	9.68	0.002**	0.21	0.648
	female	76	24	13.52	<0.001***		

* , statistical differences with *P*<0.05.

** , statistical differences with *P*<0.01.

*** , statistical differences with *P*<0.001.

**Table 3:** Results of the bioassay used to test the potential seasonal modification in *H. axyridis* sensitivity by evaluating the response to a reference winter extract of ladybeetles collected each month (n = 50 males; n = 50 females).

		Percentage of ladybeetles that selected the arm		Goodness-of-fit test statistical data		Test of independence statistical data	
Month	Sex	marked	clean	χ^2^	P	χ^2^	P
February	male	80	20	18.00	<0.001***	0.27	0.603
	female	84	16	23.12	<0.001***		
March	male	78	22	15.68	<0.001***	0.06	0.806
	female	80	20	18.00	<0.001***		
April	male	82	18	20.48	<0.001***	1.41	0.235
	female	72	28	9.48	0.002**		
May	male	60	40	2.00	0.157	0.04	0.839
	female	58	42	1.28	0.258		
June	male	42	58	1.28	0.258	0.36	0.546
	female	48	52	0.08	0.777		
July	male	52	48	0.08	0.777	0.16	0.689
	female	48	52	0.08	0.777		
August	male	42	58	1.28	0.258	1.44	0.230
	female	54	46	0.32	0.572		
September	male	52	48	0.08	0.777	1.00	0.316
	female	42	58	1.28	0.258		
October	male	70	30	8.00	0.005**	1.00	0.316
	female	66	34	5.12	0.024*		
November	male	58	42	1.28	0.258	0.04	0.839
	female	60	40	2.00	0.157		
December	male	60	40	2.00	0.157	0.39	0.534
	female	66	34	5.12	0.024*		
January	male	70	30	8.00	0.005**	0.20	0.656
	female	74	26	11.52	0.001**		

* , statistical differences with *P*<0.05.

** , statistical differences with *P*<0.01.

*** , statistical differences with *P*<0.001.

The assay used to test changes in the leading potential of the substrate marking indicated that the reared ladybeetles showed slightly positive responses toward markings collected in February, October and November. All these three markings originated from overwintering individuals. In contrast, the deposits of ladybeetles under non-aggregation conditions did not induce any preferential responses (Table 4). In June, a preference for the clean arm was even observed in females (Table 4).

**Table 4:** Results of the bioassay used to test the possible seasonal modification of the leading potential (i.e., the potential inducing a “following” response in ladybeetles) of *H. axyridis* substrate marking by evaluating the response of rearing ladybeetles toward the substrate marking of individuals collected each month (n = 50 males; n = 50 females).

		Percentage of ladybeetles that selected the arm		Goodness-of-fit test statistical data		Test of independence statistical data	
Month	Sex	marked	clean	χ^2^	P	χ^2^	P
February	male	54	46	0.32	0.572	2.06	0.151
	female	68	32	6.48	0.011*		
March	male	60	40	2.00	0.157	<0.001	>0.999
	female	60	40	2.00	0.157		
April	male	56	44	0.72	0.396	1.96	0.161
	female	42	58	1.28	0.258		
May	male	50	50	0.00	>0.999	1.01	0.315
	female	40	60	2.00	0.157		
June	male	40	60	2.00	0.157	1.10	0.295
	female	30	70	8.00	0.005**		
July	male	46	54	0.32	0.572	0.04	0.841
	female	44	56	0.72	0.396		
August	male	50	50	0.00	>0.999	1.01	0.315
	female	40	60	2.00	0.157		
September	male	42	58	1.28	0.258	1.44	0.230
	female	54	46	0.32	0.572		
October	male	64	36	3.92	0.048*	0.17	0.680
	female	60	40	2.00	0.157		
November	male	68	32	6.48	0.011*	0.40	0.529
	female	62	38	2.88	0.090		
December	male	56	44	0.72	0.369	0.04	0.840
	female	58	42	1.28	0.258		
January	male	56	44	0.72	0.369	<0.001	>0.999
	female	56	44	0.72	0.369		

* , statistical differences with *P*<0.05.

** , statistical differences with *P*<0.01.

No significant difference was observed between the behavior of males and females (Tables 2 to 4). Statistical analyses also revealed that the chemicals deposited by the ladybeetles when selecting one of the two arms did not interfere with the tested substrate marking (*P* values ranging from 0.075 to more than 0.999). Moreover, no attractive nor repellent effect of the cleaning solvent was observed for males (χ^2^ = 0.4; *P* = 0.527) or females (χ^2^<0.001; *P*<0.999).

The results obtained by performing the negative control revealed that overwintering ladybeetles that are given the choice between a clean arm and an arm coated with a substrate marking collected during summer preferentially selected the arm without any chemical compound. This behavior was observed for both sexes, whereby 64% of males (χ^2^ = 3.92; *P* = 0.048) and 68% of females (χ^2^ = 6.48; *P* = 0.011) selected the clean arm. The bioassay used to test extract collected during an aggregation period on the same overwintering ladybeetles verified that a greater proportion of individuals selected the arm presenting the winter substrate marking; whereby, 66% of males (χ^2^ = 5.12; *P* = 0.024) and 62% of females (χ^2^ = 3.27; *P* = 0.071) selected the coated arm. The independence test performed on the two data sets did not reveal any difference between the behavior of males with that of females (χ^2^ = 0.178; *P* = 0.673 for the summer substrate marking and χ^2^ = 0.221; *P* = 0.638 for the winter marking).

## Discussion

It has recently been demonstrated that substrate markings composed of alkanes and alkenes deposited by moving conspecific *H. axyridis* ladybeetles contribute to the formation of winter aggregations [2]. In this study, we highlighted that this type of substrate marking presents a chemical profile that differs according to the period of the year. We showed that markings laid by overwintering individuals contain greater proportions of di-unsaturated hydrocarbons (i.e., heptacosadiene, nonacosadiene, and hentriacontadienes) and methyl-nonacosanes, in comparison to substrate markings deposited by individuals collected outside during the summer months (i.e., April to September) (Table 1). In comparison, the opposite was observed for mono-unsaturated compounds; whereby, the proportion of nonacosene and heptacosene was greater in the substrate markings deposited by individuals collected outside compared to markings collected from overwintering ladybeetles. Furthermore, the substrate markings of individuals collected during March presented an intermediate chemical profile between the aggregation and outside groups. This difference reflects the intermediary physiological state of insects collected in March, which were captured on outside frames during their emergence from the winter aggregation. The saturated hydrocarbons were also identified as being indicator compounds of either indoor overwintering substrate markings (tricosane) or outside individual deposits (tetracosane and pentacosane) based on the statistical analyses. However, the contribution of these hydrocarbons towards differentiating the aggregation group and the outside group was less significant compared to the unsaturated compounds, with each saturated hydrocarbons being an indicator of the corresponding group in less than 40% of the cases (see the corresponding indicator values in section “Seasonal changes in the chemical profile of substrate marking”).

It is possible that the lower proportion of di-unsaturated hydrocarbons collected from outside individuals was due to the faster evaporation of these unstable compounds, or due to exposure to external conditions, such as high temperature or low humidity. The role of these physical factors has been highlighted for the cuticular profiles of two ant species, *Formica exsecta* and *Pogonomyrmex barbatus*, in which the cuticle of foragers contains a larger proportion of stable compounds compared to in-nest workers [10], [11]. However, in the current study, this hypothesis is invalid because (1) there was a greater increase in the proportions of some mono-unsaturated hydrocarbons, which are also unstable compounds, compared to the proportions of the corresponding saturated hydrocarbons [12], and (2) more than three months are required to eliminate alkadienes (mean quantity of 0.208±0.020 µg) to the point where they cannot be quantified when using the same analytical method as that described in section “Quantitative variation in chemical profile” (unpublished data). Therefore, the difference identified here is probably due to a change in the deposit of cuticular hydrocarbons.

In addition to containing different chemical profiles, substrate markings collected during the overwintering and the non-hibernating periods do not induce the same behavioral response in *H. axyridis* ladybeetles. Indeed, while most aggregating ladybeetles follow the first type of substrate markings, the same individuals are repelled by the second type of markings. It seems then that substrate markings containing greater proportions of alkadienes and methyl-nonacosanes along with lower proportions of heptacosene and nonacosene, induce a “following” cue behavior in ladybeetles searching for a place to aggregate. Alkenes often play an important role in insect communication compared to alkanes, notably in nest-mate recognition of wasps, honeybees and ants [13]–[15]. This difference might be due to alkenes having a greater number of possible configurations per chain length compared to saturated compounds, as suggested by Châline et al. [16]. Branched hydrocarbons also provide a greater number of possibilities compared to linear saturated hydrocarbons.

Chemical changes in substrate marking profile influenced ladybeetle behavior; however, other factors also appeared to be involved. Over a 1-year period, most overwintering individuals preferentially chose the arm containing conspecific deposits. Nevertheless, only weak responses were recorded in October, while no preferential response was obtained from ladybeetles collected in November. These differences might be related to the exceptional climatic conditions encountered in Belgium during these months. In October, a very exceptional sunshine period was recorded, while in November, an abnormal average temperature and exceptional sunshine period were recorded, which were all superior to normal conditions (Table 5). It is well-known that the diapause of the multicolored Asian ladybeetle during winter is regulated by photoperiod and is induced by short-day conditions [17]–[19]. The sunshine period, defined as the number of hours of bright sunshine over the month, could also be involved in the induction of this overwintering state and, consequently, influence the behavior linked to this diapause stage. In December, females only exhibited weak biological responses toward the reference winter extract and no preferential response was observed for males. Temperature might also influence overwintering behavior, as the average temperature of this month was exceptionally high compared to normal conditions, whereas the sunshine period was normal.

**Table 5:** Climatic conditions observed in Belgium during the experimental year (from February 2011 to January 2012) and typical monthly climatic values.

Month	Average temperature (°C)		Typical average temperature (°C)(a)	Sunshine period (h:min)		Typical sunshine period (h:min)(a)
February	5.4	n	3.7	54:45	n	76:36
March	7.7	n	6.8	204:13	ve	113:57
April	14.1	ve	9.8	238:51	ve	158:58
May	14.8	n	13.6	264:12	va	191:00
June	16.8	n	16.2	180:59	n	188:00
July	16	e	18.4	140:00	va	201:00
August	17.3	n	18.0	144:41	va	190:00
September	16.5	a	14.9	173:23	n	143:05
October	12.1	n	11.1	161:45	ve	113:00
November	8.6	a	6.8	114:54	e	66:17
December	6.1	e	3.9	51:56	n	45:08
January	5.1	n	3.3	48:57	n	58:34

These data were obtained from observation measurements collected at a station of the Royal Belgian Meteorological Institute (KMI-IRM) located in Uccle (45 km from Gembloux).

n, normal conditions.

a, abnormal conditions (phenomenon equal to or exceeded every 6 years).

va, very abnormal conditions (phenomenon equal to or exceeded every 10 years).

e, exceptional conditions (phenomenon equal to or exceeded every 30 years).

ve, very exceptional conditions (phenomenon equal to or exceeded every 100 years).

(a) typical values were calculated over a 30 years period which ends up the year preceding the one considered.

The multicolored Asian ladybeetle overwinters in a state of diapause, whereby it exhibits an adaptive arrest in development during an unfavorable period [20]. However, it has been demonstrated that *H. axyridis* only enters diapause for a short time, and not for the entire period that the aggregation is formed [21], [22]. After diapause, ladybeetles shift to a stage of quiescence [21]. Unlike diapause, which requires specific physiological stimuli for termination, quiescence is a stage in which insects are highly responsive to changing environmental conditions, and are able to immediately resume development and reproduction when conditions become suitable [23]. From March onwards, *H. axyridis* ladybeetles were encountered outside or walking around in the vicinity of aggregation sites, indicating that individuals collected in March had just exited the overwintering phase. However, the individuals collected in March and April positively responded to conspecific substrate markings and, more surprisingly, to the reference winter extract. As these ladybeetles had just exited the quiescent state, based on Raak-van den Berg et al. [21], individuals had already resumed normal development; hence, their behavior might not be related to aggregation any more. One explanation for this response might be the use of conspecific signals by *H. axyridis* to locate prey rapidly, and in a low-cost energy way, after depleting a large amount of energy during overwintering. Nevertheless, the use of such signals in non-social insects has never been previously considered. Therefore, further investigations are needed to understand the real motivation of this behavior.

In May and June, males also presented a “following” conspecific substrate marking behavior. This response differed to the behavior described in the preceding paragraph. First, ladybeetles followed the markings deposited by individuals in the same physiological state, but did not follow the reference winter extract. Second, only males followed the conspecific markings, with no preference being observed for females. The latter observation suggests the involvement of sexual behavior. Hemptinne et al. [24] highlighted the use of elytral cuticular hydrocarbons by males of the two-spot ladybeetle in the selection of a sexual partner. Hydrocarbons deposited on the substrate might also play a role in mate recognition in Coccinellids.

Apart from the three exceptions discussed here (males collected in May, June and December), ladybeetles collected each month exhibited a similar response toward the markings deposited by conspecifics collected at the same time and the reference winter extract. When beetles from the captive reared colony (23°C/16 h light photoperiod) were tested, weak responses toward substrate markings deposited by aggregating individuals were recorded. Moreover, no preference was observed for markings collected during the non-overwintering period, with it acting as a deterrent to females collected in June. This finding indicates that the hydrocarbon signal is more attractive when greater amounts of alkadienes and methyl-nonacosanes are present, along with weaker proportions of heptacosene and nonacosene, even for individuals that are not in the overwintering state. In conclusion, the seasonal modification observed in the “following” conspecific cues behavior arises as a result of an increase in overwintering ladybeetle sensitivity and an increase in the leading potential of the substrate marking deposited by these ladybeetles.
